# The enigma of persistent hypertriglyceridemia: A case report

**DOI:** 10.1002/ccr3.5610

**Published:** 2022-03-23

**Authors:** Armaan Dhaliwal, Soumiya Ravi, Kanwal Bains, Anil Kumar Potharaju, Tasneem Shah

**Affiliations:** ^1^ University of Arizona College of Medicine at South Campus Tucson Arizona USA; ^2^ 29761 Dayanand Medical College and Hospital Ludhiana India; ^3^ 21186 Roger Williams Medical Center Providence Rhode Island USA

**Keywords:** hypertriglyceridemia, MDPL, pancreatitis, POLD1

## Abstract

A patient with a history of Mandibular hypoplasia, Deafness, Progeroid Features Associated Lipodystrophy Syndrome (MDPL), familial lipodystrophy presented with hypertriglyceridemia induced pancreatitis with triglycerides in the 3000s. This lipodystrophy occurs due to a mutation in the POLD1 gene (DNA polymerase delta 1). MDPL, hypertriglyceridemia, pancreatitis, POLD1.

## Graphical abstract

MDPL is an extremely rare congenital disorder, which if identified can allow for the early management of metabolic disorders. Refractory hypertriglyceridemia is one of the serious features of the syndrome warranting the use of multiple medications. Patients affected by MDPL should be offered genetic counseling and close surveillance.

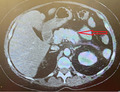

## 1 INTRODUCTION

Hypertriglyceridemia is defined as a serum triglyceride (TG) level >150 mg/dl (1.7 mmol/L). ^1^ It is commonly detected as a part of routine blood work, which includes a fasting lipid panel to assess for cardiovascular risks. It is categorized into three groups based on the triglyceride levels‐

- Normal: <150 mg/dl (1.7 mmol/L)
- Moderate hypertriglyceridemia: 150–885 mg/dl (1.7–10 mmol/L)
- Severe hypertriglyceridemia: >885 mg/dl (≥10 mmol/L)

Atherosclerotic cardiovascular diseases like myocardial infarction and cerebrovascular accidents are more common in patients with elevated fasting plasma TG levels.^2^ Hypertriglyceridemia causes about 1%–10% of acute pancreatitis cases.^3^

A less common cause of hypertriglyceridemia is lipodystrophy, which involves fat loss in a generalized or partial pattern and is often associated with hypertriglyceridemia, diabetes mellitus, and hepatic steatosis. Lipodystrophies are classified as either genetic or acquired.^4^ The acquired forms are usually caused by various infections, autoimmune diseases, and drugs such as protease inhibitors and reverse transcriptase inhibitors. There is a growing consensus among the medical community to elucidate the genetic causes of lipodystrophy. Research in the area is yielding results, but a lot remains to be comprehended. Mandibular hypoplasia, deafness, progeroid feature‐associated lipodystrophy syndrome (MDPL, MIM# 615381), and a genetic lipodystrophy are an autosomal‐dominant systemic disorder with about 25 patients reported worldwide till date.

According to a literature review, about 1000 patients have been found to have genetic lipodystrophies.^5^ Congenital generalized lipodystrophy (CGL) and familial partial lipodystrophy (FPL) are two important forms. CGL presents with overt features of lipodystrophy and can be diagnosed at birth. However, FPL is commonly misdiagnosed as metabolic syndrome in adult life due to overlapping common clinical features noticed in the two conditions. Here, we present a case of a MDPL patient presenting with hypertriglyceridemia. This report highlights the decision‐making involved in managing a patient who had refractory hypertriglyceridemia, followed by a review of genetic lipodystrophies.

## 2 CASE PRESENTATION

The patient is a 43‐year‐old woman with a past medical/surgical history significant for MDPL, diabetes mellitus, hypertriglyceridemia, hypertension, fibromyalgia, gastroesophageal reflux disease (GERD), Sjogren's syndrome, rheumatoid arthritis, irritable bowel syndrome, and cholecystectomy. Her home medications are listed in Table 1. She presented to the hospital for persistent abdominal, flank, and chest pain refractory to pain management with acetaminophen and ibuprofen at home. This pain started after she was setting decoration for the holidays. She denied any associated nausea, vomiting, fevers, chills, recent constipation, diarrhea, shortness of breath, cough, palpitations, or dysuria.

**TABLE 1:** Patient's list of home medications before admission

Metformin 1000 mg twice daily for diabetes mellitus
Glipizide 10 mg twice daily for diabetes mellitus
Pioglitazone 40 mg twice daily for diabetes mellitus
Fenofibrate 145 mg once daily for hypertriglyceridemia
Atorvastatin 40 mg once daily for hypertriglyceridemia
Lisinopril‐HCTZ 10–12.5mg once daily for hypertension
Methocarbamol 500mg twice daily for chronic back pain
Insulin Lantus 12 U once daily for diabetes mellitus
Humira 40mg once weekly for rheumatoid arthritis
Leflunomide 10 mg once daily for rheumatoid arthritis
Tramadol 50 mg once daily for chronic back pain
Pilocarpine 5 mg once daily for Sjogren
Dicyclomine 20 mg once daily for IBS
Lansoprazole 30 mg once Q Mon, Wed, Fri & Sat for GERD
Fish oil for hypertriglyceridemia

Abbreviation: GERD, Gastroesophageal reflux disease.

She denied any alcohol or recreational drug use. She quit smoking a few years back. Her mother had a history of myocardial infarction, diabetes mellitus, hypertension, and rheumatoid arthritis. Her family is originally from Mexico, and one of her brothers and her two cousins have elevated triglycerides.

Physical examination was significant for tenderness in the epigastric and bilateral costovertebral areas. Her abdomen had disproportionately increased fat deposition while her arms and legs were extremely lean (due to lipodystrophy). Her mandible was also noticed to be hypoplastic.

Her blood pressure was 125/89 mm Hg, heart rate was 140 bpm, temperature was 36.3°C, and respiratory rate was 17 breaths/minute.

The differentials based on her symptoms and physical examination included‐

- Acute coronary syndrome
- Aortic dissection
- Pulmonary embolism
- Gastroesophageal disorder
- Urinary tract infection
- Acute pancreatitis
- Musculoskeletal strain

Her CBC showed a WBC count of 11.2 k/mm^3^ with neutrophilic predominance (reference range 4–11 k/mm^3^), hemoglobin of 13.4 g/dl (reference range 12–16 mg/dl), hematocrit of 40% (reference range 35–48%), and a platelet count of 258 k/mm3 (reference range 130–450 k/mm^3^). Her urinalysis revealed the presence of blood and 3–10 RBCs/hpf.

Other pertinent laboratory results are shown in Table 2.

**TABLE 2:** Laboratory results on admission

Variable	Value	Reference range in hospital
BUN	9 mg/dl	8–25 mg/dl
Creatinine	0.35 mg/dl*	0.6–1.4 mg/dl
Sodium	127 mmol/L*	134–147 mmol/L
Potassium	4.6 mmol/L	3.5–5.3mmol/L
Chloride	89 mmol/L	95–108 mmol/L
Bicarbonate	19 mmol/L	19–31 mmol/L
Glucose	271 mg/dl*	70–106 mg/dl
Calcium	9.3 mg/dl	8.8–10.4
Total protein	7.5 g/dl	6–8 g/dl
Albumin	3.8 g/dl	3.4–4.9 g/dl
Total bilirubin	0.5 mg/dl	0.2–1.3 mg/dl
AST	40 U/L	10–41 U/L
ALT	20 U/L	5–46 U/L
ALP	30 U/L	37–127 U/L
Troponin	8 ng/L	<11 ng/L
Lactic Acid	2.1 mmol/L	0.5–2.2 mmol/L
D‐dimer	537*	<500
Total cholesterol	452 mg/dl*	<199 mg/dl
HDL	8 mg/dl*	>50mg/dl
Triglycerides	3154 mg/dl*	<149 mg/dl
Lipase	172 U/L*	16–63 U/L
TSH	3.52 uIU	0.45–4.5 uIU

* It signifies abnormal values

**FIGURE 1:**
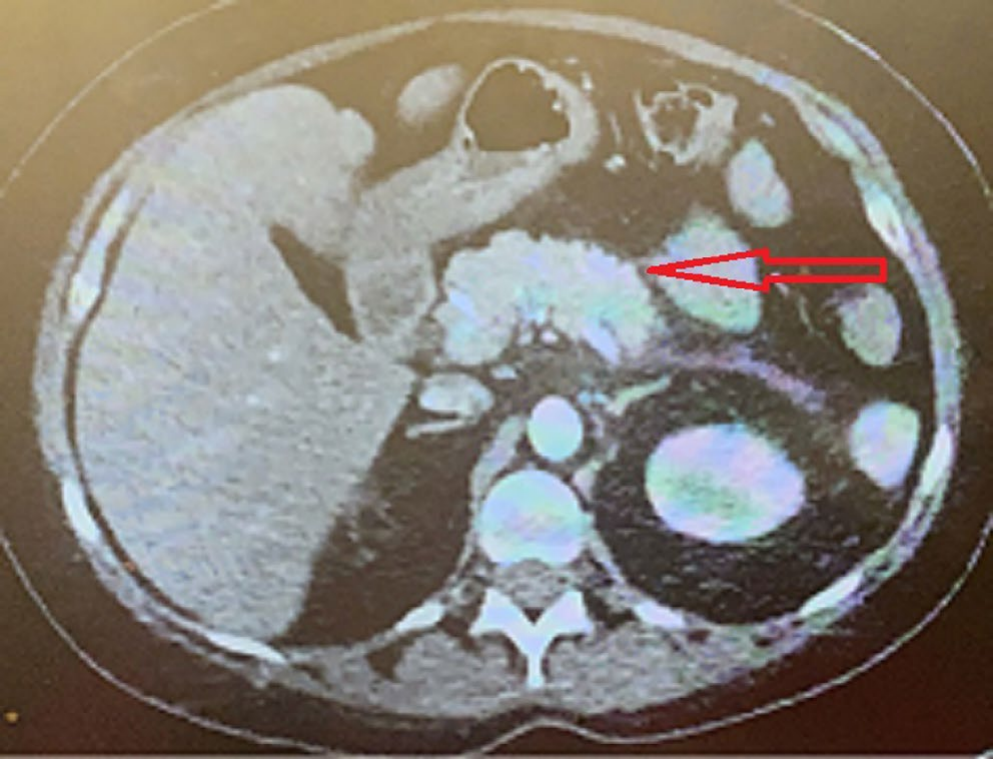
CT showing an inflamed and edematous pancreas as depicted by the arrow, concerning for pancreatitis

In the emergency department, she received 1 L of normal saline and 4 mg of IV morphine for pain control. Her chest X‐ray did not show any acute cardiopulmonary process. Her low troponin and the absence of EKG changes rendered acute coronary syndrome unlikely. Her urinalysis was negative for an infection. A CT angiogram of the chest and abdomen was performed: This ruled out any aortic pathology and PE but was positive for acute pancreatitis as depicted in Figure 1. The patient met all three criteria of acute pancreatitis with elevated lipase levels, epigastric pain, and imaging findings of pancreatitis with elevated triglycerides confirming the diagnosis of acute pancreatitis secondary to hypertriglyceridemia. She was admitted for further management. She was kept nil per mouth, and her pain was managed with oxycodone and morphine. She was able to consume a clear liquid diet a day after her presentation and was easily transitioned to a regular diet.

To lower her triglycerides, we started an insulin drip at a rate of 5 units/h. Dextrose 5% half‐normal saline with 40 mEq/L of potassium was initiated at 250 ml/h; insulin and glucose checks every 1 h were requested for monitoring. Her electrolytes and triglycerides were monitored every four hours. After one day of being on an insulin infusion, she was transitioned to subcutaneous glargine insulin 20 units daily.

However, 8 h after transitioning to subcutaneous insulin, her triglycerides started to rise again requiring reinitiation of IV insulin. On this occasion, she was started on 20 units of subcutaneous glargine with 8 units of lispro. In addition, we restarted her fenofibrate 160 mg daily and increased her statin to 80 mg daily. Her triglycerides eventually dropped below 1000 in a day but fluctuated at levels over 500. Subsequently, she was discharged on glargine, fenofibrate, atorvastatin, and fish oil with her triglyceride levels in the 500s with close outpatient endocrinology follow‐up. A further decline in triglycerides was not pursued due to concerns for hypoglycemia and low incidence of hypertriglyceridemia induced pancreatitis at levels in the 500s.

Her leflunomide for rheumatoid, pilocarpine for sjogren, lansoprazole for GERD, and dicyclomine for irritable bowel syndrome were resumed at discharge.

**FIGURE 2:**
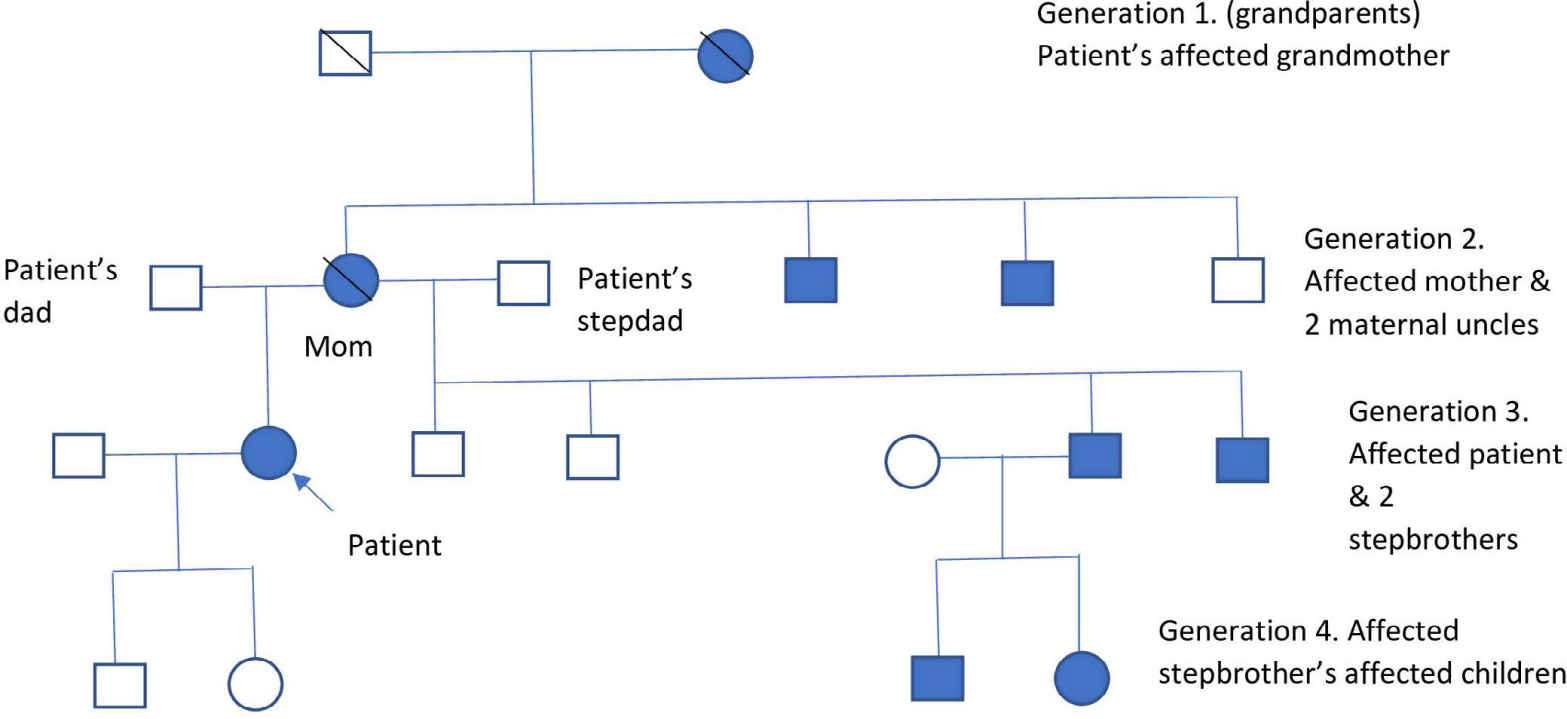
Family pedigree of the case. Her mother had lipodystrophy and died at age 68 years due to a myocardial infarction but no information about her father, a visiting Austrian. The patient also had an unaffected stepdad. She also has two maternal uncles with lipodystrophy. Two out of her four stepbrothers have lipodystrophy, and one also has crowded teeth. One affected stepbrother also has 2 children with lipodystrophy with no genetic evaluation. Her grandmother had similar features and died at age of 80 years. She has a mixed European and Native American ancestry with no Jewish heritage and consanguinity. Her cousin and nephew also had a history of mandibular hypoplasia, deafness, progeroid features, and lipodystrophy syndrome (MDPL). Shaded shapes‐ affected with MDPL or some form of lipodystrophy, clear shapes unaffected. Generation 1‐ grandparents, generation 2‐ parents, dad, stepdad and maternal uncles, generation 3‐ patient, husband, and stepbrothers, and generation 4‐ patient's and stepbrother's children

The patient presented to our facility for the first time in 2016 for lipodystrophy with a history of diabetes mellitus and menstrual cramps. POLD 1 (DNA polymerase delta 1) mutation was suspected given a positive family history. Her family pedigree is represented in Figure 2. She was tested for POLD1 gene mutation, which revealed the presence of a variant of uncertain significance (UVUS), c.1519C>T (p. Arg507Cys) in POLD1, prompting further evaluation of her condition. This sequence change replaces arginine with cysteine at codon 507 of the POLD1 protein. The arginine residue is highly conserved, and there is a sizeable physiochemical difference between arginine and cysteine. This variant was not found to be in the population databases at that time.

## 3 DISCUSSION

### 3.1 Mandibular hypoplasia, deafness, progeroid feature‐associated lipodystrophy syndrome

Most patients initially present with lipodystrophy and distinctive facial features like prominent eye, beaked nose, small mouth, and overcrowded teeth. Premature aging accompanied by sensorineural hearing loss and conjunctival telangiectasia helps to differentiate MDPL from other progeroid syndromes. In contrast to other progeroid disorders, MDPL itself does not predispose to accelerated atherosclerosis.^6^ Due to the missense mutation in the proofreading domain of POLD1, there is a theoretical predisposition for colorectal cancer.^7^ But to date, none of the MDPL patients developed colorectal cancer. Men display cryptorchidism and hypogonadism, with certain women presenting with menstrual irregularities like amenorrhea and dysmenorrhea. Managing hypertriglyceridemia can be cumbersome because of the use of medications like statins and fibrates together leading to increased risk of drug induced myopathies. Proper management of comorbidities like diabetes mellitus and hypertriglyceridemia can vastly improve the survival in MDPL, and these patients can have a normal life expectancy under close surveillance.

The presence of concomitant autoimmune disorders warrants attention because of their direct and indirect associations with hypertriglyceridemia. Two cases of Sjogren‐like syndrome have been reported secondary to increased triglycerides, which resolved after management of hypertrigylceridemia.^8^ Leflunomide, commonly used in the management of rheumatoid arthritis causing severe hypertriglyceridemia, has also been reported.^9^ Our case's Sjogren symptoms did not improve with resolution of hypertriglyceridemia. Her hypertriglyceridemia can be explained by her MDPL, but future episodes of hypertriglyceridemia‐induced pancreatitis on maximal treatment require a discussion about choosing an alternative to leflunomide. Patient was also on hydrochlorothiazide, known to elevate cholesterol levels and lead to increased triglycerides in 5%–15% of the cases.^10^ The exact mechanism leading to elevated triglycerides is unknown. An alternate antihypertensive should be considered in in patients at risk of developing hypertriglyceridemia.

Mutations in the polymerase delta (POLD 1) gene located on chromosome locus 19q13, which performs DNA synthesis in the lagging strand during DNA replication that is responsible for MDPL presentation.^7^ Heterozygous de novo mutations in the POLD 1 gene with most cases involving single‐codon deletion (p. S605del) lead to the decreased catalytic activity of POLD 1 enzyme with a partially altered proofreading activity, making it the deletion hot spot for MDPL. Some cases have been reported to have a missense mutation (p.R507C) replacing arginine with cytosine, as observed in the presented case.

### 3.2 Mandibuloacral hypoplasia‐associated lipodystrophy

Mandibuloacral hypoplasia‐associated lipodystrophy (MAD) is a rare autosomal‐recessive systemic disorder that presents with lipodystrophy, growth retardation, skeletal abnormalities, mandibular hypoplasia, and mottled cutaneous pigmentation. The absence of diabetes mellitus helps to differentiate between MDPL and MAD.^11^

### 3.3 Werner syndrome

Werner syndrome (adult progeria), an autosomal‐recessive disorder, is characterized by premature aging with other age‐related diseases, short stature, and bilateral cataracts.^12^

### 3.4 Congenital generalized lipodystrophy

About 300 patients suffer from congenital generalized lipodystrophy (CGL) characterized by near total lack of body fat with significant muscularity recognized at birth or soon thereafter. There are four different phenotypes of CGL (CGL 1–4) caused by mutations in different genes.^4^

CGL1‐ Loss of body fat
CGL2‐ Loss of body fat, mild mental retardation, and cardiomyopathy
CGL3‐ Loss of body fat, short stature, and vitamin D resistance
CGL4‐ Loss of body fat, myopathy, pyloric stenosis, and cardiomyopathy

### 3.5 Proteasome‐associated autoinflammatory syndrome

Proteasome‐associated autoinflammatory syndrome (PRAAS) is an autosomal‐recessive disorder that presents with partial lipodystrophy, plaques on the face and extremities, basal ganglia calcifications, and joint contractures.^13^

Other entities that fall into the category of molecular lipodystrophies include.

- Short stature, hyperextensibility of joints and/or inguinal hernia, ocular depression, Rieger anomaly and teething delay syndrome (SHORT)
- Neonatal progeroid syndrome/Wiedemann‐Rautenstrauch syndrome (WR)
- Marfanoid‐progeroid‐lipodystrophy syndrome (MPL)

## 4 CONCLUSIONS

Hypertriglyceridemia can be associated with acquired and congenital lipodystrophies. One of the types of congenital lipodystrophy syndromes is mandibular hypoplasia, deafness, and progeroid feature‐associated lipodystrophy syndrome (MDPL). Recognizing the phenotypical features of the syndrome and diagnosing the same can help in making appropriate lifestyle decisions. It should lead to more frequent check‐ups to monitor the development of diabetes mellitus and hypertriglyceridemia‐associated complications. Identifying the POLD1 gene mutation can be important for genetic counseling and clinical surveillance in different clinical avenues including hearing, cardiovascular, and cancer predisposition.

## CONFLICT OF INTEREST

None.

## AUTHOR CONTRIBUTIONS

Armaan Dhaliwal managed the patient, collected data, did research, and wrote manuscript. Soumiya Ravi did research and wrote manuscript. Kanwal Bains did research and wrote manuscript. Anil Kumar Potharaju involved in correction of the manuscript. Tasneem Shah managed the patient and involved in correction of the manuscript.

## ETHICAL APPROVAL

This case report was approved by the Banner University Medical Center, South Campus ethics committee. A written consent to publish the case report was also obtained from the patient.

## CONSENT

A written consent to submit and publish the report was obtained from the patient prior to submission.

## Data Availability

Data sharing is not applicable to this article as no new data were created or analyzed in this study.
